# On the neural implementation of the speed-accuracy trade-off

**DOI:** 10.3389/fnins.2014.00236

**Published:** 2014-08-13

**Authors:** Dominic Standage, Gunnar Blohm, Michael C. Dorris

**Affiliations:** ^1^Department of Biomedical and Molecular Sciences, Queen's UniversityKingston, ON, Canada; ^2^Institute of Neuroscience, Shanghai Institutes for Biological Sciences, Chinese Academy of SciencesShanghai, China

**Keywords:** speed-accuracy trade-off, decision making, neural mechanisms of cognition, bounded integration, review

## Abstract

Decisions are faster and less accurate when conditions favor speed, and are slower and more accurate when they favor accuracy. This phenomenon is referred to as the speed-accuracy trade-off (SAT). Behavioral studies of the SAT have a long history, and the data from these studies are well characterized within the framework of bounded integration. According to this framework, decision makers accumulate noisy evidence until the running total for one of the alternatives reaches a bound. Lower and higher bounds favor speed and accuracy respectively, each at the expense of the other. Studies addressing the neural implementation of these computations are a recent development in neuroscience. In this review, we describe the experimental and theoretical evidence provided by these studies. We structure the review according to the framework of bounded integration, describing evidence for (1) the modulation of the encoding of evidence under conditions favoring speed or accuracy, (2) the modulation of the integration of encoded evidence, and (3) the modulation of the amount of integrated evidence sufficient to make a choice. We discuss commonalities and differences between the proposed neural mechanisms, some of their assumptions and simplifications, and open questions for future work. We close by offering a unifying hypothesis on the present state of play in this nascent research field.

## 1. Introduction

The ability to trade-off speed and accuracy against each other is a hallmark of decision making across species and tasks (Chittka et al., 2009; Bogacz et al., 2010a; Heitz and Schall, 2012). For a given task difficulty, decisions are typically faster and less accurate when conditions favor speed, and are slower and more accurate when conditions favor accuracy. Given the near-ubiquity of this behavior in experiments, the speed-accuracy trade-off (SAT) can almost be considered a psychophysical law. It can also be considered a cognitive phenomenon, since it captures a change in strategy toward an ostensibly unchanging task.

The SAT has long been the subject of behavioral experiments (Fitts, 1966; Wickelgren, 1977), but studies addressing its neural basis are a fairly recent development in the field of decision making (Bogacz et al., 2010b). These studies have built on a large body of work on the neural basis of decisions more generally. This work has characterized the computations underlying decisions (see Smith and Ratcliff, 2004; Ratcliff and McKoon, 2008), identified neural correlates of these computations (see Schall, 2001; Gold and Shadlen, 2007; Kable and Glimcher, 2009) and provided mechanistic hypotheses that explain behavioral data in terms of neural data (see Wang, 2008, 2012). This body of work provides a persuasive account of neural decision processing, but does not speak directly to the mechanisms by which decision processing is differentially modulated by conditions favoring speed or accuracy.

In this review, we describe hypotheses on the neural implementation of the SAT. We take a modeling perspective. We classify models according to two general levels of abstraction, sometimes referred to as the algorithmic level and the level of implementation (Marr, 1982). These classes need not be considered discrete, but rather, can be considered as the extreme ends of a continuum. At one end, algorithmic models characterize the computations underlying brain function. At the other end, neural models address the implementation of these computations. In the domain of decision making, analytic studies have shown the assumptions and constraints under which implementation-level models are formally equivalent to algorithmic models, providing a principled foundation for considering the latter in terms of the former (Bogacz et al., 2006). We endeavor to utilize the flexibility and explanatory power of this modeling perspective.

Our review is structured according to the framework of bounded integration. This framework not only provides a set of organizing principles for the review, but provides the background for this collection more generally. Most of the neural and behavioral data we consider were recorded from perceptual decision tasks. We assume that the neural mechanisms underlying perceptual decisions generalize to other kinds of decisions, but the sources of evidence differ according to the decision domain (Gold and Shadlen, 2007). Sensory systems and memory systems provide examples of sources of evidence. We begin by defining the SAT (Section 2). We then describe bounded integration as a computational framework for characterizing decisions (Section 3), along with a widely held hypothesis on the neural implementation of these computations (Section 3.1). We categorize hypotheses on the SAT according to the major components of the bounded integration framework, describing the evidence for differential modulation of these components under speed and accuracy conditions (Sections 4.1, 4.2, and 4.3). We close with a discussion of the assumptions underlying these hypotheses, the relationship between mechanisms, and some open questions for future research (Section 5).

## 2. Defining the speed-accuracy trade-off

**Figure 1:**
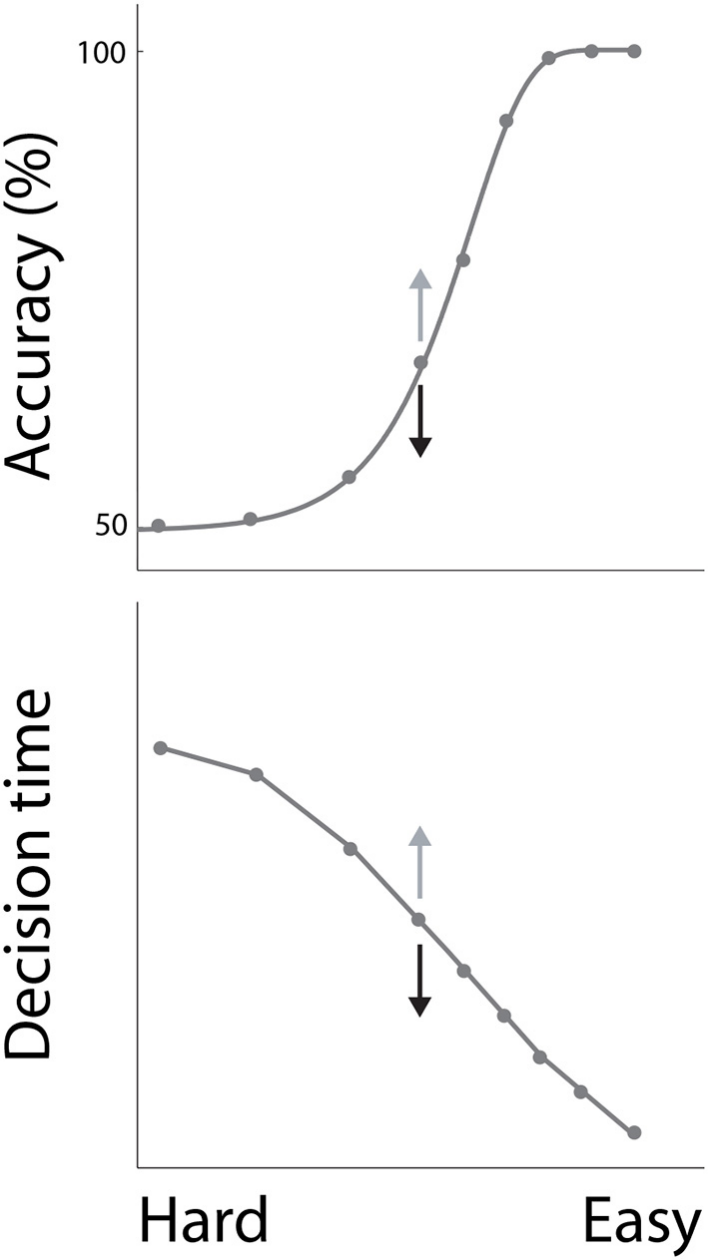
**The psychometric and chronometric curves**. Decisions are faster and less accurate with increasing task difficulty, describing a relationship between speed and accuracy. For a given task difficulty, decisions are faster and less accurate under conditions favoring speed, and are slower and more accurate under conditions favoring accuracy. This phenomenon is depicted by the arrows on either side of the central data point on each curve, where speed and accuracy conditions correspond to black and gray arrows respectively.

In decision tasks, subjects must determine which decision alternative is favored by the evidence. If the evidence for one alternative is clearly stronger than the evidence for the others, the task is easy. Conversely, if the evidence for each alternative is similar, the task is difficult. Accuracy decreases with task difficulty, while decision times increase, characterizing the common psychometric and chronometric curves respectively (Figure 1). Task difficulty therefore imposes a systematic relationship between the speed and accuracy of decisions (see Stone, 2014 in this collection), but these curves do not define the SAT. The SAT refers to changes in the speed and accuracy of decisions *for a given task difficulty*. While many decision tasks manipulate the strength of evidence, this experimental parameter need not vary in SAT experiments.

The SAT captures a control mechanism for decision processing, and can be further distinguished according to the timescale of adjustments to speed and accuracy conditions. Over longer timescales, the SAT may be accomplished by adaptive mechanisms that extract a balance between the speed and accuracy of decisions in order to maximize reward over a block of trials (Gold and Shadlen, 2002; Simen et al., 2006; Furman and Wang, 2008; Standage et al., 2011). This approach has been demonstrated by algorithmic models (Gold and Shadlen, 2002; Bogacz et al., 2010a), biophysically-based neural models (Lo and Wang, 2006; Furman and Wang, 2008) and models in between these levels of abstraction (Simen et al., 2006). In contrast, experimental subjects often learn to respond to speed or accuracy conditions from trial to trial, according to a pre-trial cue (Forstmann et al., 2008; Heitz and Schall, 2012). We point out this difference because we are unaware of any implementation-level models to simulate trial-to-trial switching of response “modes” for speed and accuracy. Since there is an optimal trade-off for *each* condition that depends on its associated reward schedule, it is plausible that long-timescale mechanisms correspond to a learning phase for each response mode; however, it is important to note that switching between speed and accuracy modes necessarily involves additional mechanisms to associate the cues with the appropriate mode, and to switch between modes on cue.

## 3. The bounded integration framework

Under the bounded integration framework, the evidence for each alternative of a decision is integrated until the running total for one of the alternatives reaches a criterion level. Thus, the bound refers to the criterion and integration refers to the accumulation of evidence. The accumulated evidence for a given alternative is referred to as a decision variable. According to this sequential sampling approach (see Ratcliff and Smith, 2004; Smith and Ratcliff, 2004), integration is necessary because neural processing of the evidence is noisy, as may be the evidence itself. By integrating the evidence over time, an average is computed, so that decisions are not based on moment-to-moment fluctuations in the evidence or its processing. The longer the integration period, the better the average and the higher the probability of identifying the alternative with the most evidence. Clearly, speed and accuracy make conflicting demands under this framework.

**Figure 2:**
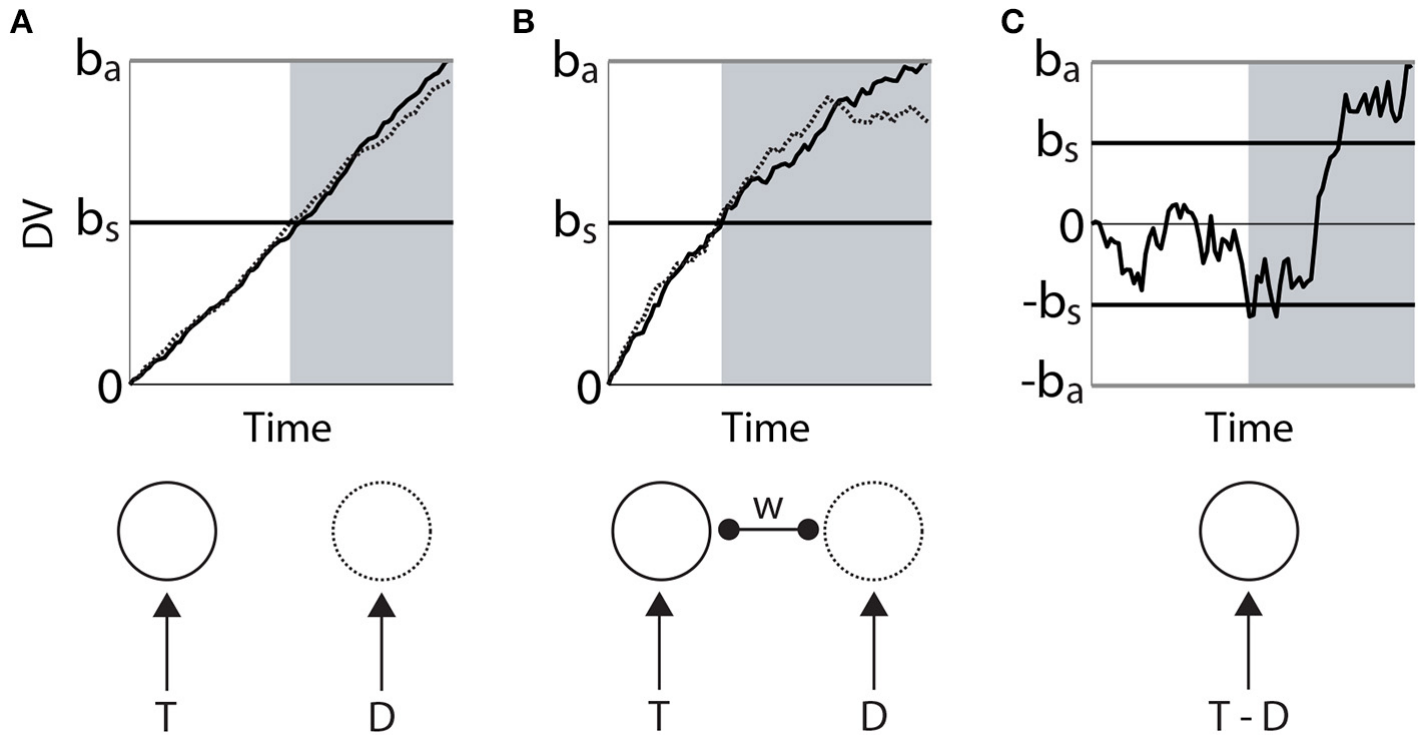
**Three classes of bounded integrator model**. Each model receives the same two noisy stimuli, one with a higher mean (target T, solid) than the other (distractor D, dotted). Curves in the upper figures correspond to integrators (decision variables DV), depicted in the lower figure. **(A)** Independent race model of a 2-choice decision. The black horizontal line *b*_*s*_ corresponds to a low decision bound, supporting faster decisions that are less likely to identify the target. The gray horizontal line *b*_*a*_ corresponds to a higher bound, favoring the accurate identification of the target at the expense of processing time. The independence of the integrators is depicted in the lower figure. **(B)** Competing accumulator model. The weight (w) of subtraction between the two integrators is depicted in the lower figure. Different values of this weight would yield different curves. **(C)** Drift diffusion model. The decision variable is the integrated difference between the two stimuli. Black (*b*_*s*_) and gray (*b*_*a*_) horizontal lines correspond to bounds favoring speed and accuracy respectively. In each panel, the gray shaded region depicts the time of crossing of the lower (speed condition) threshold.

Bounded integration subsumes a number of algorithmic models. Most generally, these models can be distinguished according to whether the evidence for each choice is integrated independently from the others, or whether the evidence for each choice serves as evidence against the others. The former are often referred to as race models (Figure 2A) and the latter as diffusion models (Figure 2C). A flexible approach between these extremes is provided by competing accumulator models (Usher and McClelland, 2001; Bogacz et al., 2007; Purcell et al., 2012), in which decision variables for the respective alternatives are subtracted from one another according to a scaling parameter or *weight* (Figure 2C). In 2-choice tasks, the weight of subtraction can effectively (though not always formally) interpolate between the independent race model and the diffusion model, i.e., it controls the strength of competition between accumulators. Moreover, competing accumulators accommodate tasks with any number of choices and they provide an important link between models at the algorithmic level and the implementation level (see the next section). For an intuitive description of the formal relationships between race models, diffusion models and competing accumulators, see Bogacz (2007). For a rigorous mathematical treatment, see Bogacz et al. (2006).

This brief description of bounded integration warrants several technical points. Firstly, integration refers to the accumulation of evidence in continuous time, but for simplicity, we do not distinguish accumulation in discrete time from the continuous-time case. Secondly, the benefits of integration depend on the timescale of noise correlations. Thirdly, we only consider unbiased tasks, in which the bound (or its mean) is the same for each alternative, as is the starting value (or its mean) of each decision variable. Note that “unbiased” does not imply that the mean evidence for each alternative is equal, but rather, the prior probability of each alternative is equal. The framework is readily extended to biased conditions (see Gold and Shadlen, 2001). For a comprehensive description of bounded integration, see Smith and Ratcliff (2004); Bogacz et al. (2006).

### 3.1. Interpreting bounded integrator models

As noted in the Introduction, bounded integrator models can be thought of as abstract algorithms that characterize the computations underlying decisions. From this perspective, the terms and parameters of these models are independent of their implementation and do not require explicit neural interpretation. On the other hand, it can be instructive to interpret these parameters in neural terms if they resemble neural activity. As such, the evidence in perceptual decision tasks corresponds to the response by sensory (and sensory-association) neurons to task-relevant stimuli, and decision variables correspond to the activity of downstream neural populations hypothesized to integrate this activity. Accordingly, the starting point of a decision variable is commonly equated with the baseline (pre-trial) level of integrator activity and the bound is commonly equated with the level of this activity at the time of commitment to a choice (see Bogacz et al., 2010b).

There is considerable evidence supporting this general interpretation. For example, in random dot motion (RDM) tasks, subjects are rewarded for identifying the direction of coherent movement of a proportion of randomly moving dots on a computer screen. The coherence of the dots provides the evidence in the task, which can be precisely controlled by the experimenter. Neurons in the medial temporal area (MT) of monkeys are responsive to movement of the dots (Britten et al., 1992, 1993), and in tasks in which monkeys indicate their choices by making an eye-movement to a visual target, neurons in the lateral intraparietal area (LIP) that are responsive to the chosen target (target-in neurons) show buildup activity prior to choice selection (Roitman and Shadlen, 2002; Churchland et al., 2008). Since MT projects to LIP, it is widely believed that neurons in LIP integrate the evidence provided by MT, projecting in turn to the circuitry mediating eye-movements (see Gold and Shadlen, 2007; Shadlen and Kiani, 2013). Note that neural correlates of decision variables in RDM tasks have also been recorded in other cortical areas, e.g., dorsolateral prefrontal cortex (dlPFC) (Kim and Shadlen, 1999) and the frontal eye fields (FEF) (Ding and Gold, 2012). Similar data have been recorded from these and other brain regions in different task paradigms, described below in relation to SAT experiments. Importantly, electrophysiological recordings from neurons responsive to a visual target that is *not* chosen on a given trial (target-out neurons) typically show a much lower rate of activity than target-in neurons prior to choice selection (e.g., Roitman and Shadlen, 2002; Thomas and Pare, 2007; Bollimunta and Ditterich, 2011; Ding and Gold, 2012). Taken together, increasing activity by target-in neurons and suppressed activity by target-out neurons have been interpreted as revealing competitive interactions between neural decision variables (Usher and McClelland, 2001; Wang, 2002; Albantakis and Deco, 2009; Standage and Pare, 2011). In competing accumulator models, each accumulator can be thought of as a population of neurons responsive to one of the alternatives, where the weight of subtraction corresponds to the strength of inhibition between these populations (Figure 2B).

Competing accumulator models can also have parameters governing leakage and recurrent excitation of decision variables, both of which are important for interpreting these models in neural terms. To begin with, neurons leak, e.g., membrane potential and synaptic activation decay. Importantly, the relevant time constants of decay (e.g., the time of decay from maximum to half-maximum) are on the order of tens of milliseconds, whereas perceptual decision times are typically in the range of several hundreds of milliseconds. Thus, the time constants of these currents are not long enough to support temporal integration. Such long integration times are believed to require recurrent excitation (Wang, 2002), provided by synaptic connectivity within a population of excitatory neurons responsive to a given alternative. To provide an idealized example, if the leakage and inhibitory synaptic currents of individual neurons (responding linearly to their inputs) were precisely offset by the strength of recurrent excitation from other neurons in the population, then each neuron would support perfect integration of evidence, limited only by its maximum firing rate. In reality, local-circuit dynamics constrain the length of time each population can support integration, described in the next section.

This neural interpretation of competing accumulator models sets the stage for our consideration of the neural basis of the SAT. In bounded integrator models, we interpret noisy evidence as the response by populations of sensory (and sensory-association) neurons to stimuli in perceptual tasks. We interpret temporal integration as the buildup activity of neural populations receiving projections from sensory neurons. We interpret the starting point of a decision variable as the activity of integrator populations at the time of evidence onset (the baseline rate), and for simplicity, we interpret the bound as the rate of integrator activity at the time of commitment to a choice. We consider another interpretation of the bound in Section 4.2.1.

#### 3.1.1. Attractor dynamics

**Figure 3:**
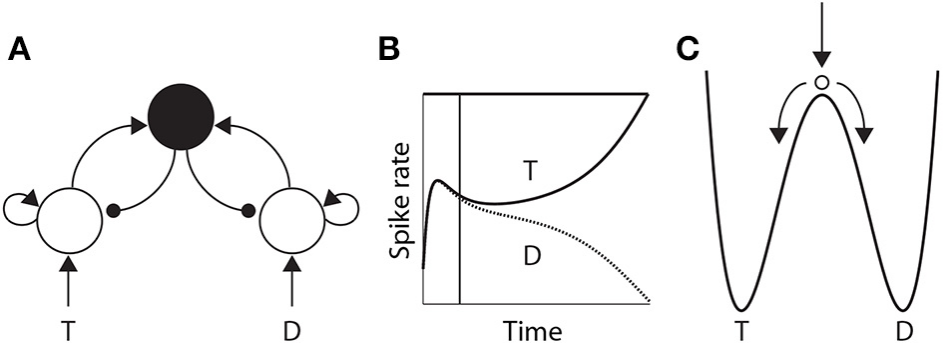
**(A)** A neural implementation of the principles of bounded integration. Neural populations selective for the decision alternatives (T and D) compete via a common inhibitory pool (solid black circle). Arcs with arrows and filled circles depict excitatory and inhibitory synaptic connectivity respectively. Target (T) and distractor (D) stimuli provide stronger and weaker evidence to the integrator populations respectively. **(B)** Competitive interactions between integrator populations lead to an increased spike rate by one population (solid) and a decreased rate by the other (dotted). **(C)** Cartoon depiction of an attractor “energy landscape” supported by the neural model, where the energy decreases over time. An unstable steady state (high energy) separates two attractors (low energy), corresponding to the target and the distractor. The ball depicts the state of the network, which is drawn toward the unstable steady state at stimulus onset (vertical arrow), and from which it is repelled toward one of the “attractor basins” (bent arrows). Descent into the attractor basin corresponds to the firing-rate excursion of the target population in **(B)**, where the vertical line approximates the position of the ball in **(C)**. The evolution of the network state (conceptually, the movement of the ball) is faster (slower) under speed (accuracy) conditions.

The time over which competing neural populations can integrate evidence is an emergent property of network dynamics. The relevant dynamics are most easily described for 2-choice decisions, but are applicable to more than two decision alternatives (You and Wang, 2013). As noted above, when the activity of an integrator population builds up in a 2-choice task, it suppresses the other population by recurrent inhibition. The eventual state of high-rate activity by one population and low-rate activity by the other is an attractor in the space of possible states of the network, and the increase in activity by the “winning” population and the suppression of the losing population (Figure 3B) corresponds to a descent into its basin of attraction (Figure 3C). The attractors are stable states of the network, that is, the state of the network evolves toward these states for a given set of conditions. Once there, the mean activity of the network is fixed until conditions change, such as the offset of evidence. In the domain of decision making, the “getting there” is the decision process.

The attractors are separated by an *unstable* steady state, toward which the network is drawn with the onset of the evidence, and from which it is repelled toward one of the two attractors (Figure 3C). The dynamics in the vicinity of the unstable steady state are slow, supporting temporal integration. The time over which integration is supported is referred to as the *effective time constant* of the network, and corresponds to the rate at which the dynamics evolve near this state. See Wong and Wang (2006) for a thorough description of the dynamics. The crucial point here is that the effective time constant is shorter with stronger recurrent dynamics, limiting the amount of time the network can integrate evidence. Accordingly, moderate dynamics can be considered to support neutral conditions, where stronger and weaker dynamics support speed and accuracy conditions respectively. We refer to local-circuit dynamics with these properties as the “decision regime.” We refer to weaker dynamics without these properties as the “leakage regime.” In the leakage regime, the effective time constant of the network is similar in principle to the time constant of decay of membrane potential or synaptic activation, though it can be considerably longer. In the decision regime, the effective time constant does not correspond to leakage; rather, it corresponds to an amplification of the decision variable, and is thus qualitatively different than a time constant of decay (see Standage et al., 2011).

## 4. Three general mechanistic hypotheses on the SAT

Hypotheses on the neural implementation of the SAT must provide mechanistic explanations for differential decision processing under speed and accuracy conditions. Under the principles of bounded integration, these hypotheses can be grouped into three mutually-compatible classes: modulation of the encoding of evidence, modulation of the integration of encoded evidence, and modulation of the amount of integrated evidence sufficient to make a choice. In principle, each class of hypothesis (and each mechanistic hypothesis in each class) is sufficient to account for the SAT, but we do not favor any one hypothesis over the others. Rather, we believe the SAT is likely to result from the interplay of multiple mechanisms, with different mechanisms (or combinations of mechanisms) playing a greater role in different contexts.

**Figure 4:**
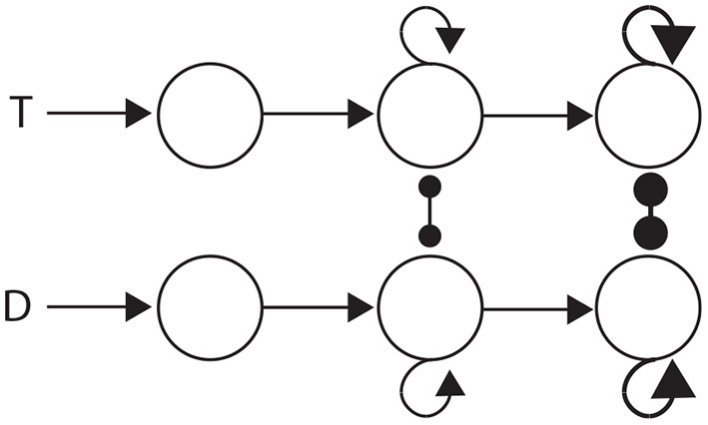
**Three processing stages for decisions: the encoding of evidence (left), the integration of encoded evidence (middle) and choice selection (right)**. Evidence-encoding populations **(left)** are responsive to target (T) and distractor (D) stimuli. Weak dynamics prevent integration, depicted by the lack of recurrent connectivity. Evidence-encoding populations project to integrator populations **(middle)**. Feedback connectivity depicts moderately strong dynamics, suitable for temporal integration (corresponding to Figure 3). Integrator populations project to thresholding circuitry **(right)**. Thick connectivity depicts very strong dynamics, suitable to an all-or none response to a critical level of input (see Simen, 2012).

The three general classes of hypothesis provide an intuitive basis for organizing the review, but they also correspond to three successive processing stages of decisions: the encoding of evidence, the integration of encoded evidence, and choosing. Under the attractor framework, the computational requirements of these stages are supported by weak, moderate and strong local-circuit dynamics respectively. Weak dynamics support the encoding of evidence by “giving way” to their inputs, i.e., the dynamics are dominated by leakage. Moderately strong dynamics furnish a long effective time constant, supporting temporal integration (Section 3.1.1). Strong dynamics furnish a short effective time constant within the decision regime, allowing an all-or-none response to a critical level of input (see Simen, 2012). Thus, the principles of bounded integration are captured by a three-stage neural system, in which evidence-encoding circuitry with weak dynamics projects to integrator circuitry with moderate dynamics, which in turn projects to thresholding circuitry with strong dynamics. This three-stage process is depicted in Figure 4.

Finally, it is important to clarify our usage of several terms before proceeding with the review. We define the “correct” alternative as the one for which the evidence has the highest mean, and as suggested in Section 2, we define task difficulty as the difference between the mean of the evidence for the correct alternative and that for the alternative with the next highest mean. Task difficulty overlaps with the rate of integration in bounded integrator models, but this overlap depends on model specifics. For example, in race models, increasing the evidence for the correct alternative increases its integration rate (there's more instantaneous input to accumulate) and reduces task difficulty if the evidence for the other alternatives is not increased; however, increasing the evidence for each alternative by the same amount increases the integration rate of each integrator, but does not influence task difficulty. In diffusion models, an increase in the evidence for the correct alternative necessarily decreases task difficulty, unless the signal-to-noise ratio (SNR) of the evidence is preserved. Here, it is important to remember our definition of the SAT in Section 2: improvements in speed (accuracy) at the expense of accuracy (speed) for a *given* task difficulty. In Section 4.2.1, we describe hypotheses on the neural implementation of the SAT by modulation of the rate of integration. We define the rate of integration as the inverse of the difference between the rate of integrator neurons at the time of commitment to a choice and their baseline rate. These considerations highlight two important points. Firstly, the hypotheses in Section 4.2.1 do not refer to changes in integration rate resulting solely from upstream changes to the encoding of evidence (support for this possibility is described in Section 4.1). Secondly, these hypotheses address the neural mechanisms by which the rate of rise of putative integrator activity is modulated by speed and accuracy conditions, not task difficulty.

### 4.1. Modulation of the encoding of evidence

**Figure 5:**
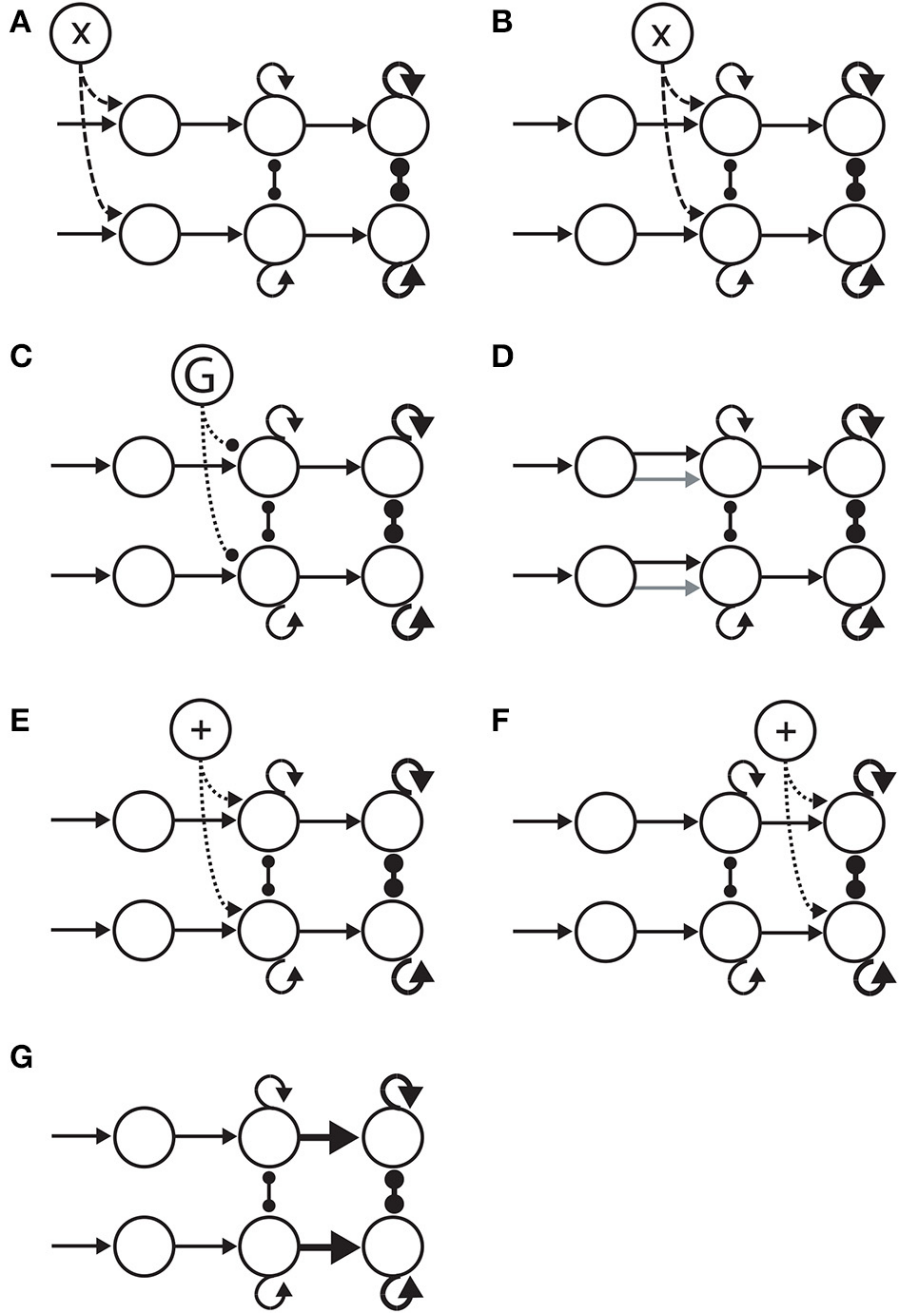
**Hypotheses on the neural implementation of the SAT, under the framework of bounded integration**. Each panel is an instance of the 3-stage schematic in Figure 4. **(A)** Modulation of the encoding of evidence. A cognitive signal adjusts the gain of sensory encoding populations (dashed arcs). This multiplicative effect is depicted by the “X” in the open circle at the top. **(B)** Modulation of the rate of integration of encoded evidence (dashed arcs). The cognitive signal adjusts the gain of integrator circuitry, controlling the rate of integration. **(C)** Modulation of the onset of integration of encoded evidence. An inhibitory gate (G) controls the onset of integration (dotted arcs). **(D)** Modulation of the sensitivity of integrator circuitry to encoded evidence. Integrator populations are selective for different sub-populations of evidence-encoding neurons under speed and accuracy conditions, depicted by the black (speed) and gray (accuracy) arcs. **(E)** Modulation of the amount of non-evidence input to integrator circuitry. All integrator populations receive a uniform cognitive signal, in addition (+) to evidence (dotted arcs). **(F)** Modulation of the amount of non-integrator input to thresholding circuitry. Neural populations enacting choice behavior receive a uniform cognitive signal, in addition (+) to integrated evidence (dotted arcs). **(G)** Modulation of the connectivity between integrator circuitry and thresholding circuitry. The amount of integrated evidence sufficient to make a choice is modulated by the strength of connectivity from integrators to the circuitry enacting choice behavior (thick horizontal arrows).

**Figure 6:**
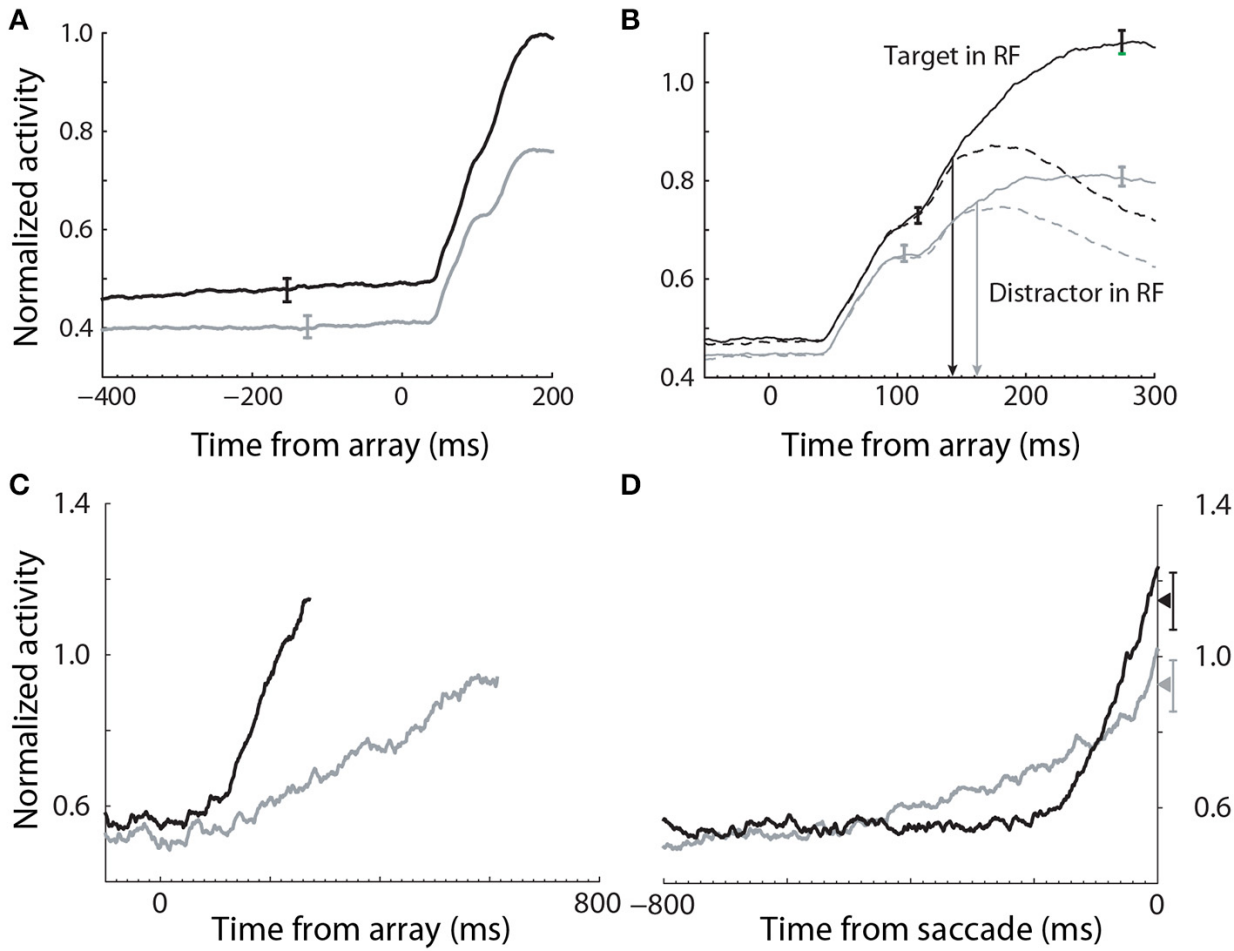
**Electrophysiological data recorded from FEF during a visual search task under speed (black) and accuracy (gray) conditions (Heitz and Schall, 2012)**. **(A)** The baseline rate of visual (evidence-encoding) neurons was higher and lower under speed and accuracy conditions respectively (stimulus onset at 0 ms). **(B)** The gain of visual neurons was higher (lower) under speed (accuracy) conditions. **(C)** The slope of movement neuron activity was higher (lower) under speed (accuracy) conditions. **(D)** The peak of movement neuron activity was higher (lower) under speed (accuracy) conditions. Data aligned to saccade initiation. Figure adapted from Heitz and Schall (2012) with permission of © Elsevier.

Evidence for the modulation of sensory processing under speed and accuracy conditions (Figure 5A) has been shown in a visual search task, in which monkeys were rewarded for making a saccade to a target stimulus, while single-cell activity was recorded from FEF (Heitz and Schall, 2012). A substantial body of electrophysiological data from visual decision tasks indicates that FEF neurons can be classified as visual neurons and movement neurons (Cohen et al., 2010; Purcell et al., 2010). Visual neurons are responsive to task-relevant stimuli, but do not show saccade-related activity, whereas movement neurons show saccade-related activity, but do not respond to stimuli. As such, movement neurons are hypothesized to integrate the evidence encoded by visual neurons (the first and second stages of Figure 4), loosely analogous to the hypothesis that LIP neurons integrate the activity of MT neurons in RDM tasks (Section 3.1). In the study by (Heitz and Schall, 2012), the SAT was correlated with multiple adjustments to the activity of both classes of neuron, including the baseline rate of visual neurons (Figure 6A), the magnitude of their response to stimuli (Figure 6B) and the time at which target-in activity can be discriminated from target-out activity (Figure 6B). To summarize, the search array was identical across conditions, but the baseline rates and response magnitude of visual neurons were higher, and the time of discrimination was earlier, under the speed condition, in which the monkeys made faster, less accurate decisions. Conversely, baseline rates and response magnitude were lower, and discrimination was later, under the accuracy condition, in which the monkeys made slower, more accurate decisions.

These data provide strong support for the hypothesis that the modulation of the encoding of evidence contributes to the SAT, but the data alone do not explain the underlying neural mechanism. Gain modulation provides an explanation. The baseline rates of target-in *and* target-out visual neurons were higher (lower) under speed (accuracy) conditions (solid and dashed curves before stimulus onset in Figure 6B), suggesting that visual neurons received a common signal, regardless of whether they were encoding evidence for the target or a distractor. Spatially non-selective (global, uniform, diffuse) excitation is an established form of gain modulation in attractor models (Salinas and Abbott, 1996; Furman and Wang, 2008; Standage et al., 2013), so a stronger (weaker) common signal under speed (accuracy) conditions would account for the higher (lower) response magnitude of visual neurons. If the SNR of encoded evidence were unaffected (or lowered) by this signal, then other things being equal, higher-rate activity by visual neurons under the speed condition would be manifest in a higher rate of integration of this activity by movement neurons, supporting fewer sequential samples and therefore improved speed at the expense of accuracy (*vice versa* for the accuracy condition). This scenario is equivalent to adjusting a decision bound. Consistent with this possibility, the rate of rise of movement-neuron activity was higher (lower) under the speed (accuracy) condition in the study by Heitz and Schall (2012). In the next section, we provide another, compatible explanation of these movement-neuron data.

### 4.2. Modulation of the integration of encoded evidence

Mechanistic hypotheses on the trading of speed and accuracy by modulation of the integration of evidence can be grouped into three mutually compatible categories: modulation of the rate of integration (Figure 5B), modulation of the onset of integration (Figure 5C) and modulation of the sensitivity to the encoding of evidence (Figure 5D). As noted above, the first hypothesis does not refer to changes in the rate of integration resulting solely from changes in the evidence or its encoding. Rather, we refer to mechanisms hypothesized to actively target integrator circuitry in this section, regardless of upstream or downstream modulation.

#### 4.2.1. Modulation of the rate of integration of evidence

The study by Heitz and Schall (2012) not only provides evidence for the differential modulation of sensory encoding with speed and accuracy conditions, but also for the modulation of the rate of integration of evidence (Figure 5B). In their study, the slope of pre-saccadic activity by movement neurons in FEF was shown to increase and decrease under speed and accuracy conditions respectively (Figure 6C). As noted in Section 4.1, these changes could simply result from the increase (decrease) in gain of visual neurons under speed (accuracy) conditions; however, they can be explained by the modulation of local-circuit (recurrent) dynamics (Figure 3), independent of upstream changes. Increasing the strength of recurrent dynamics shortens the effective time constant of local-circuit models (Wong and Wang, 2006; Standage et al., 2011), so the decision variable builds up more quickly, limiting the amount of integrated evidence. Decisions are consequently faster and less accurate. Conversely, decreasing the strength of recurrent dynamics lengthens the effective time constant, so the decision variable builds up more slowly and decisions are slower and more accurate. Here, it is worth noting that the computational role of the effective time constant is identical to that of the bound, operating at a different level of abstraction; it controls the duration of the integration of evidence. Thus, while it is intuitive to interpret the bound in terms of the firing rates of integrator neurons, the bound may be implemented by any mechanism that controls integration time.

Lengthening and shortening the effective time constant of a decision circuit offers a sound principle for trading speed and accuracy, but it requires a mechanism (or mechanisms) to increase and decrease the strength of recurrent dynamics under speed and accuracy conditions respectively. There are several possibilities, such as spatially non-selective excitation of excitatory neurons (Furman and Wang, 2008; Standage et al., 2013) or the conductance strength of excitatory recurrent synapses (Wong and Wang, 2006; Standage and Pare, 2011). Furman and Wang (2008) used the first of these mechanisms in simulations of an RDM task with a biophysically-based local-circuit model. They simulated the experiments by Churchland et al. (2008), who recorded from LIP neurons while monkeys chose between two or four possible directions of motion. Not only did Furman and Wang (2008) qualitatively reproduce neural and behavioral data from the task, but they further considered the effects of speed and accuracy emphasis that were not tested experimentally. They hypothesized that the SAT is controlled by a stationary “top-down” signal, testing their hypothesis by providing non-selective spike trains to all pyramidal neurons in the network, in addition to the selective spike trains simulating motion evidence from area MT. Stronger non-selective input produced faster, less accurate decisions in the model. Furman and Wang (2008) did not show network activity under the different non-selective input rates, but it is clear from other modeling studies that the slope of network activity is higher (lower) with stronger (weaker) recurrent dynamics, corresponding to speed (accuracy) emphasis (e.g., Wong and Wang, 2006; Standage and Pare, 2011). Notably, the baseline rates of target-in *and* target-out movement neurons in the electrophysiological study by Heitz and Schall (2012) were higher (lower) under speed (accuracy) conditions, consistent with the modulation of local-circuit dynamics by a spatially non-selective signal. Note that such a signal is consistent with the use of the term “urgency” in some studies, i.e., speed (accuracy) conditions entail a higher (lower) urgency to respond (Reddi and Carpenter, 2000), though we restrict our usage of this term to time-dependent signals below, i.e., the urgency to respond increases with the duration of a decision (Churchland et al., 2008; Cisek et al., 2009; Standage et al., 2011).

**Figure 7:**
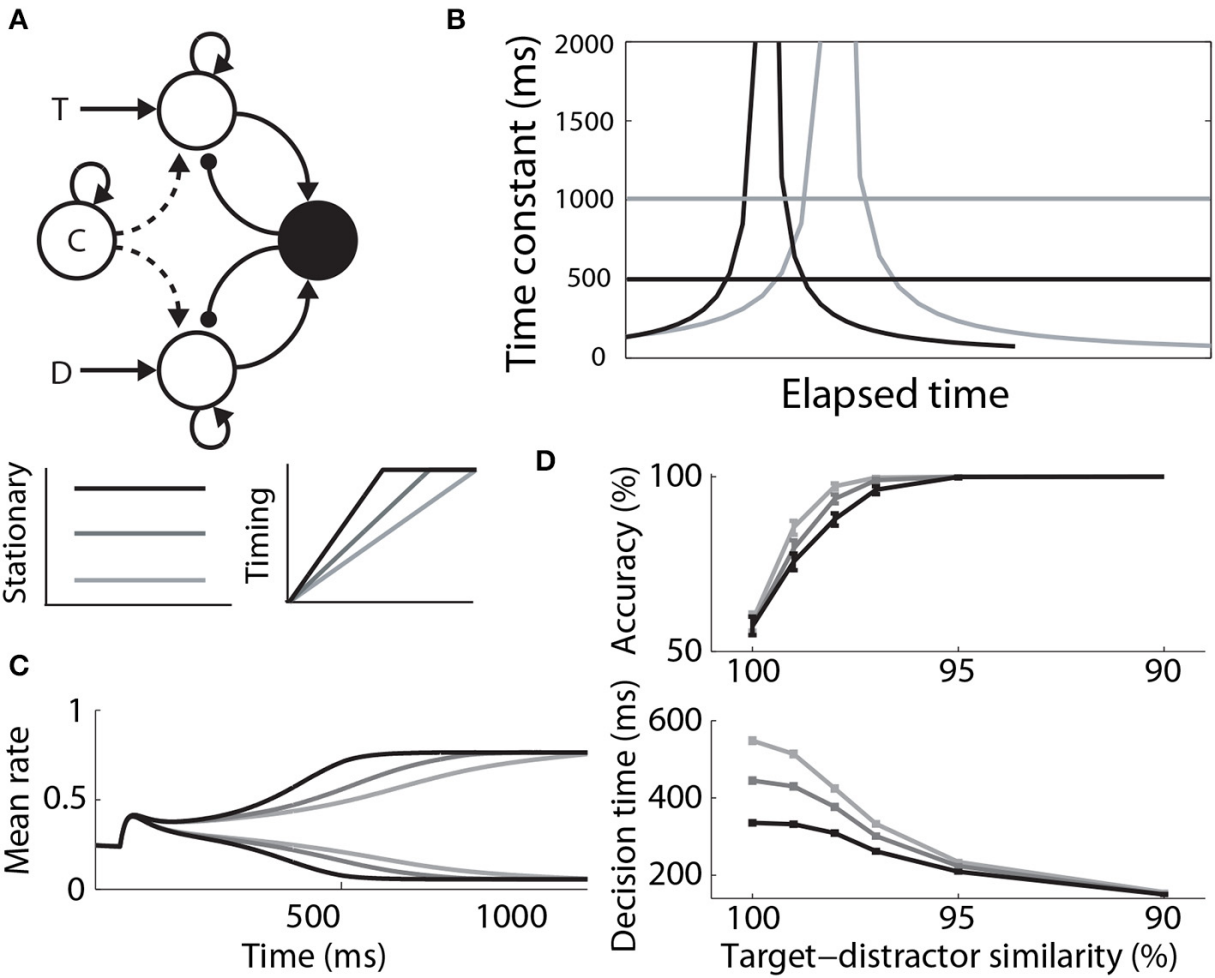
**(A)** A control signal (c) modulating local-circuit decision processing. The signal could be implemented by persistent, goal directed activity (Stationary) or by climbing activity, encoding elapsed time relative to a deadline (Timing). The model schematic is the same as in Figure 3A, with the addition of the control signal. **(B)** Under stationary modulation, the decision network can support a single time constant of integration for a given trial, depicted by the horizontal lines. A stronger control signal furnishes a shorter time constant. Under time-dependent modulation, the time constant of integration increases in the leakage regime, before contracting in the decision regime. This progression occurs more quickly with faster buildup of the signal. Black and gray curves correspond to speed and accuracy conditions. **(C)** Target and distractor-selective firing rates in a simulated decision circuit for each timing signal. The slope of decision-selective activity is higher for shorter timing signals. **(D)** Psychometric and chronometric curves corresponding to each timing signal. **(B–D)** are adapted from Standage et al. (2011).

Where Furman and Wang (2008) used a stationary signal to differentially modulate decision dynamics under speed and accuracy conditions, Standage et al. (2011) used a timing (urgency) signal, hypothesizing that an estimate of one's temporal constraints is sufficient to trade speed and accuracy with a fixed level of integrator activity at decision time. They used a model from the same family as that of Furman and Wang (2008), but they took a more abstract *population rate* approach, where a “transfer function” determines the proportion of an idealized neural population activated by its input (Wilson and Cowan, 1972; Gerstner, 2000). The timing signal was an increasing function of time, building up more quickly with tighter temporal constraints, but reaching a fixed maximum (see Durstewitz, 2004). The signal scaled the slope parameter of the transfer function, which in turn controlled the dynamics of the network (the higher the slope parameter, the stronger the dynamics). As such, network dynamics were weak at the start of each trial, but were strengthened with elapsed time. This progression lengthened the time constant of the network prior to entry into the decision regime, and then shortened it (Figure 7B). Decision-selective firing rates were fixed at decision time because the network always progressed through the same dynamic regimes, but slower buildup of the timing signal allowed the network to spend more time in regimes with a longer time constant. Thus, the slope of integrator activity was lower (higher) with longer (shorter) temporal constraints, and decisions were slower (faster) and more (less) accurate (Figures 7C,D). Standage et al. (2011) compared this approach to the modulation of the network by a stationary signal, showing that time-dependent modulation systematically earned more reward per unit time. In effect, time-dependent modulation of attractor dynamics makes a better use of time than stationary modulation, but human and non-human animals do not necessarily make decisions this way. The model makes testable predictions for experiments, which are an important next step for this hypothesis (see the Discussion).

What neural mechanisms could implement stationary (Furman and Wang, 2008; Roxin and Ledberg, 2008) and time-dependent (Standage et al., 2011, 2013) top-down signals for controlling the speed and accuracy of decisions? A stationary signal could be provided by persistent, goal-directed activity, for which there is abundant evidence in prefrontal and parietal cortical areas (see Wang, 2001). This mechanism would require an additional means to control the rate of persistent activity. Like integration time, the rate of persistent activity in local-circuit cortical models can be controlled by the strength of recurrent dynamics (Brunel and Wang, 2001). Thus, any mechanism that modulates recurrent dynamics in the circuitry mediating the control signal would in turn control the strength of non-selective input to downstream integrator circuitry, and thereby the SAT. To switch between speed and accuracy response modes from trial to trial (e.g., Forstmann et al., 2008; Heitz and Schall, 2012), higher and lower rates of persistent activity would need to be associated with the cues for speed and accuracy conditions respectively.

There is also abundant evidence for the encoding of elapsed time by “climbing activity,” i.e., activity that peaks at the time of an anticipated event, such as a deadline (see Durstewitz, 2004). Such *prospective coding* (Rainer et al., 1999; Komura et al., 2001) has been recorded during tasks with a timing requirement in a number of cortical areas (Niki and Watanabe, 1979; Rainer et al., 1999; Maimon and Assad, 2006; Shuler and Bear, 2006). Standage et al. (2013) built on their earlier population rate model (Standage et al., 2011) with a biophysically-based, coupled-circuit cortical model, offering a neural implementation of the timing signal, and demonstrating its modulation of downstream decision dynamics by spatially non-selective excitation. To switch between speed and accuracy response modes from trial to trial, the shorter and longer timing signals would need to be associated with the cues for speed and accuracy conditions respectively.

It is worth noting that time-dependent attractor models Standage et al. (2011, 2013) are conceptually similar to bounded integrator models in which the bound is lowered over the course of each trial (Ditterich, 2006b; Drugowitsch et al., 2012), but the former cannot be considered a neural implementation of the latter. The underlying premise of the latter is that longer processing time implies a more difficult decision and therefore a lower probability of a correct response. Lowering the bound reduces time-wasting because it speeds up decisions that are more likely to be wrong, increasing reward rate. This approach is functionally equivalent to the time-dependent multiplication of incoming evidence (Ditterich, 2006b). Expressed as a bounded integrator model, the time-dependent attractor models by Standage et al. (2011, 2013) implement the time-dependent multiplication of evidence *and* the evolving decision variable, making different predictions about the sensitivity of decisions to the timing of evidence than other bounded integrator models (see Section 5.1).

#### 4.2.2. Modulation of the onset of integration

It is possible that speed and accuracy conditions modulate the onset of evidence integration (Figure 5C), as opposed to (or in addition to) the rate of integration. Purcell et al. (2012) tested this hypothesis with a leaky competing accumulator model, in which the accumulators received the activity of visually-responsive neurons in FEF, recorded during a visual search task. The accumulator corresponding to the target received the activity of target-in neurons, while the other accumulators received the activity of target-out neurons. Each accumulator received a fixed inhibitory signal serving as a gate, preventing the accumulation of activity prior to the search array, that is, the gate dictated that evidence was only accumulated if it exceeded a minimum rate. The model was fit to behavioral data from monkeys performing the search task and to electrophysiological recordings from FEF movement neurons. In simulations of an SAT experiment, adjustments to the inhibitory gate were compared to adjustments to the bound. Both parameters accounted for the SAT and maximized reward rate, but they made different predictions about the activity of movement neurons. As expected, adjustments to the bound predicted a higher (lower) rate of activity at the time of commitment to a choice under accuracy (speed) conditions, but did not impact baseline activity or the onset of integration. Adjustments to the inhibitory gate predicted higher (lower) baseline activity and earlier (later) onset of integration under speed (accuracy) conditions. To the best of our knowledge, the activity of FEF movement neurons in the study by Heitz and Schall (2012) provide the only available single-cell data to test these predictions. These data do not support the predictions of the bound parameter. Not only do they show differential baseline activity under speed and accuracy conditions, but they also show a higher rate of activity at choice time under speed conditions (Figure 6D), i.e., opposite to the predicted activity. These data support the predictions for baseline activity by the gate parameter, i.e., higher baseline under speed conditions, but they do not support the prediction of differential onset of integration. Several fMRI studies with human subjects also show differential baseline activity under speed and accuracy conditions in pre-motor cortical areas (Forstmann et al., 2008; Ivanoff et al., 2008; van Maanen et al., 2011) (Section 4.3.2).

#### 4.2.3. Modulation of the sensitivity to encoded evidence

**Figure 8:**
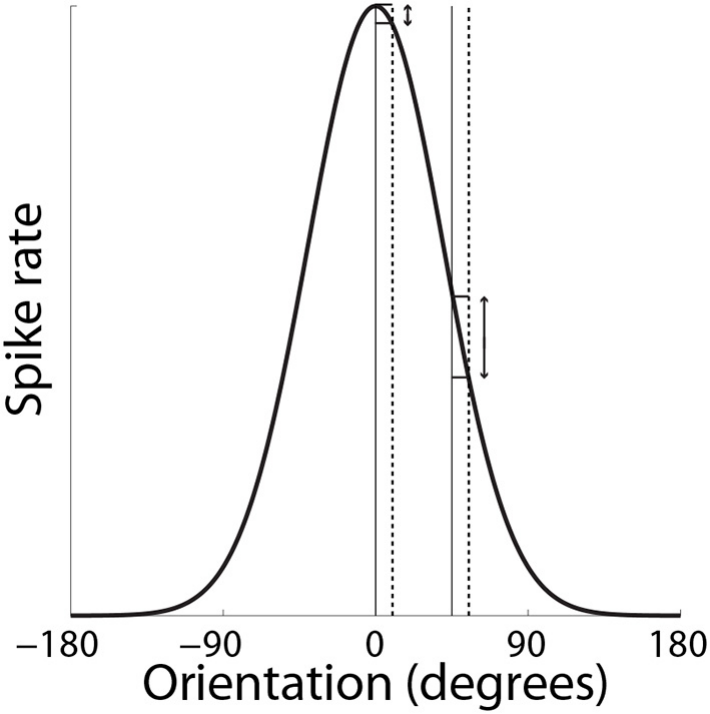
**SAT mechanism hypothesized by Ho et al. (2012)**. Small changes to a stimulus feature do not elicit much change in the response by neurons that are highly selective for the feature (on-target neurons). Here, the feature is orientation. The solid and dashed vertical lines on the left correspond to feature values of 0° and slightly greater than 0° respectively. The change in response by a neuron maximally responsive to 0° is shown by the corresponding horizontal lines abutting the black curve. The solid and dashed vertical lines on the right correspond to feature values of 45° and a slight increase from 45° respectively. The change in response by the same neuron (maximally responsive to 0°) is shown by the corresponding horizontal lines abutting the black curve. For a given change in feature value, the difference in the off-target response is greater than the difference in the on-target response.

Support for the hypothesis that integrator circuitry is more (less) sensitive to the encoding of evidence under accuracy (speed) conditions (Figure 5D) has been provided by a visual discrimination task, in which human subjects decided whether flashing stimuli were of the same or slightly different orientation (Ho et al., 2012). As expected, decisions were slower and more accurate under the accuracy condition (*vice versa* for speed). Because the neural mechanisms underlying fine discrimination of orientation are well-studied, these authors focused on trials on which the stimuli differed (mismatch trials). In particular, off-target neurons (tuned away from the stimulus) are hypothesized to be more informative for fine discrimination than on-target neurons (tuned toward the stimulus), due to the steeper slope of their tuning curves at off-target orientations (see Scolari and Serences, 2012). This computational principle is depicted in Figure 8. In the study by Ho et al. (2012), there was no difference between blood oxygenation level dependent (BOLD) based orientation tuning curves in primary visual cortex (V1) under speed and accuracy conditions, suggesting that these conditions did not modulate the encoding of evidence on mismatch trials. However, off-target activation (tuned away from the target orientation) was higher on correct trials than error trials under the accuracy condition, that is, subjects were more accurate when off-target activation was higher. This finding suggests that subjects were more accurate when the gain of off-target neurons was higher, which further suggests that accuracy was higher because integrator populations detected this higher gain. Conversely, BOLD-based tuning curves did not differ on correct and error trials under the speed condition, suggesting that integrator populations did not detect fluctuations in the gain of off-target neurons (or on-target neurons). Taken together, the speed and accuracy data suggest that integrator populations are more sensitive to (more informative) off-target activity under accuracy conditions, resulting in higher accuracy at a cost in terms of speed. Under speed conditions, lower sensitivity to off-target activity would appear to support faster decisions, at a cost in terms of accuracy.

Ho et al. (2012) did not speculate on the mechanism by which speed (accuracy) conditions may engender lower (higher) sensitivity to more informative neurons, but it is plausible that speed conditions lower the SNR of the activity projecting to integrator circuitry, such that the fine discrimination provided by off-target activity is swallowed by noise. The lower firing rate of off-target activity (see Figure 8) is consistent with this possibility. Another possibility is that integrator circuitry is not differentially sensitive to off-target activity *per se*, but is preferentially *selective* for on-target and off-target neurons under speed and accuracy conditions respectively. If so, lower-rate, more informative off-target activity would take longer to accumulate to a given firing rate than higher-rate, less informative on-target activity, accounting for the SAT. Our description of this possibility does not explain how preferential selectivity would arise, but is consistent with the higher (lower) rate of rise of movement-neuron activity under speed (accuracy) conditions shown by Heitz and Schall (2012).

### 4.3. Modulation of the amount of integrated evidence sufficient to make a choice

The hypothesis that speed and accuracy are traded by the modulation of the amount of integrated evidence has received the lion's share of attention in mechanistic studies of the SAT, presumably because bounded integrator models are readily fit to behavioral data by adjusting the bound (see Bogacz et al., 2010b). Under the assumption of linear integration, changing the starting point is algorithmically equivalent to changing the bound. Under a neural instantiation of these terms, changes to the starting point would be manifest in changes to the baseline activity of integrator neurons, while changes to the bound would be manifest in the firing rate of integrator neurons at the time of commitment to a choice. Here, it is important to distinguish between the amount of integrated *evidence* and a neural decision variable. A decision variable may have sources of input other than the evidence (Kable and Glimcher, 2009; Doya and Shadlen, 2012), e.g., the encoding of the prior probabilities of the alternatives. Under this approach, mechanistic hypotheses on the modulation of the amount of integrated evidence sufficient to make a choice can immediately be grouped into two categories: changes to non-evidence inputs to integrator circuitry (Figure 5E), and changes to non-integrator inputs to thresholding circuitry (Figure 5F). The former tend to be limited to cortical circuitry, whereas the latter often involve cortex and the basal ganglia (BG). We also consider a third category in this section: changes to the connectivity mediating integrator inputs to thresholding circuitry (Figure 5G). This category is distinct from the modulation of integrated evidence described above (Section 4.2), since no mechanistic change to the integration process is entailed by changes to downstream connectivity. Note that these three general, mechanistic categories share the assumption that a fixed net input current to thresholding circuitry is required to elicit choice behavior.

#### 4.3.1. Adjustments to non-evidence inputs to integrator circuitry

Several theoretical studies have proposed neural mechanisms for the SAT that involve differential levels of non-evidence inputs to integrator circuitry under speed and accuracy conditions (Furman and Wang, 2008; Roxin and Ledberg, 2008; Standage et al., 2013) (Figure 5E). A large body of electrophysiological data provides evidence for integrator activity in frontal (Kim and Shadlen, 1999; Schall et al., 2011; Ding and Gold, 2012) and parietal (Roitman and Shadlen, 2002; Thomas and Pare, 2007; Bollimunta and Ditterich, 2011) cortical areas during decision tasks (Section 3.1), so these theoretical studies have typically focused on cortical circuitry. Furman and Wang (2008) controlled the SAT by providing input spike trains to all pyramidal neurons in their biophysically-based cortical model, in addition to the selective spike trains for each of the decision alternatives. We presented this model in Section 4.2.1 because spatially non-selective input modulates the dynamics of local-circuit decision models, changing the rate of integration. However, the model does implement an adjustment to the amount of non-evidence input to integrator circuitry, albeit a small one.

The hypothesis that persistent activity controls the SAT by projecting non-selectively to integrator populations (Furman and Wang, 2008; Roxin and Ledberg, 2008) is consistent with fMRI data from a Simon task (van Veen et al., 2008), in which human subjects responded to the color of a stimulus to the left or right of fixation, while ignoring its location. This study showed an increased baseline (sustained) BOLD response in dlPFC under speed conditions relative to accuracy conditions, and an increased transient (associated with the decision process) BOLD response in the intraparietal lobule, a parietal area that may correspond to LIP in monkeys. As noted above, persistent activity has been recorded from dlPFC in studies of working memory (Fuster, 1973; Funahashi et al., 1989) and decision-correlated activity has been recorded from LIP in decision tasks (Roitman and Shadlen, 2002; Thomas and Pare, 2007), so it is plausible that dlPFC projects a stronger (weaker) control signal to integrator neurons in the intraparietal lobule under speed (accuracy) conditions, controlling the speed and accuracy of decisions. This possibility is consistent with increased (decreased) baseline activity by putative integrator neurons under speed (accuracy) conditions in the study by Heitz and Schall (2012) (Section 4.2.1), as well as with the modulation of the rate of integration by a stationary, non-selective signal (Furman and Wang, 2008).

The SAT is also controlled by non-selective excitation of integrator circuitry in the model by Standage et al. (2013). As described in Section 4.2.1, the major difference between this neural model and the one by Furman and Wang (2008) is the information content of the non-evidence input. In the model by Standage et al. (2013), the non-evidence input is an estimate of elapsed time relative to a deadline, implemented by the destabilization of background activity by strong recurrent dynamics. Like the model by Furman and Wang (2008), this model controls the SAT by modulation of the rate of integration (Section 4.2.1), but nonetheless, it does implement a time-dependent, uniform input to integrators. This input builds up more (less) rapidly under speed (accuracy) conditions.

#### 4.3.2. Adjustments to non-integrator inputs to thresholding circuitry

**Figure 9:**
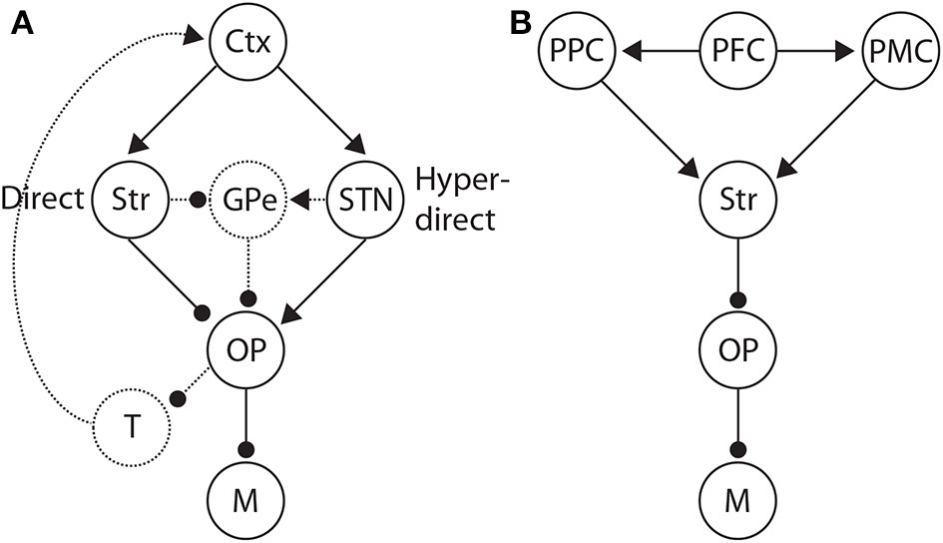
**(A)** Basal ganglia (BG) pathways hypothesized to control the SAT. Along the direct pathway, cortex (Ctx) excites the striatum (Str), which in turn inhibits the BG output nuclei (OP). The output nuclei project tonic inhibition to the circuitry driving motor execution of decisions (M), i.e., choices. Less integrator activity is required to make a choice when the striatum is diffusely excited by non-integrator cortical activity. Along the hyper-direct pathway, cortex excites the subthalamic nucleus (STN), which in turn excites the output nuclei. More integrator activity is required to make a choice when STN is diffusely excited by non-integrator cortical activity. The dotted arcs through the globus pallidus (GPe) depict two further pathways that could influence the SAT in an opposite manner to the direct and hyperdirect pathways respectively. Routes back to cortex via the thalamus (T) are also depicted by dotted arcs, largely unexplored in this context. **(B)** Distributed system of brain regions correlated with decision making and the SAT. Data and theory suggest that executive cortical areas (here PFC) project non-selectively to integrator populations (here posterior parietal cortex PPC) and to pre-motor areas (PMC). A stronger (weaker) non-selective signal thus favors speed (accuracy) by increasing (decreasing) the strength of recurrent dynamics among integrator populations (Figure 3B) and by decreasing the rate of their activity required for choice behavior.

A number of mechanistic hypotheses on the SAT are based on the premise that the amount of integrated evidence sufficient to make a choice is controlled by spatially non-selective input to thresholding circuitry (Frank, 2006; Simen et al., 2006; Forstmann et al., 2010; Green et al., 2012) (Figure 5F). According to this premise, stronger non-selective input allows lower levels of integrator activity to elicit a choice. The hypotheses differ according the processing pathways providing the non-selective inputs, and in the corresponding information content provided by these signals. Many of these hypotheses involve BG, owing to its well-established role in movement initiation (choice behavior in the present context). Excitatory input to BG arrives at the striatum, which inhibits the output nuclei along the so-called direct pathway. The output nuclei inhibit motor circuitry in their tonic (background, default) state, so excitation of the striatum releases motor circuitry from inhibition, enabling choice behavior (See Figure 9A).

It has been proposed that an estimate of reward rate could provide spatially non-selective input to thresholding circuitry, computed by leaky integration of reward signals (Simen et al., 2006). Such a mechanism could approximate the optimal trade-off between speed and accuracy in terms of reward-rate maximization, without speed or accuracy instructions (Simen et al., 2006). In effect, the strength of non-selective input tracks reward rate under this mechanism. It is plausible that such a non-selective signal could be implemented in PFC by the increased occupancy of D1 dopamine receptors, due to slow extrasynaptic uptake (Grace, 1991; Dreyer et al., 2010). The activity of dopamine (DA) neurons in BG is extensively correlated with reward and these neurons project diffusely to PFC (and other association cortical areas), where D1 receptors are hypothesized to control attractor dynamics in support of persistent, goal-directed activity (see Durstewitz and Seamans, 2006). It is therefore possible that the rate of persistent activity in PFC could provide a reward estimate to BG, which gates choice behavior. It is not clear how such a reward-rate signal would adapt to the imposition of speed or accuracy conditions on cue, i.e., the proposed mechanism extracts an appropriate strength of signal for a given condition, but would presumably require an additional mechanism to switch between speed and accuracy modes from trial to trial.

Timing signals are another potential source of non-selective input to thresholding circuitry. Under this hypothesis, the SAT is controlled by the balance between selective input from integrator populations and non-selective input from neural populations encoding elapsed time. In the study by Green et al. (2012), human subjects performed an RDM task under reward schedules corresponding to speed and accuracy conditions. Subjects' behavior was fit by a bounded integrator model, where adjustments to the bound were correlated with reward rate on an individual subject basis, i.e., subjects whose behavior was captured by larger adjustments to the bound earned more reward. Because a higher (lower) bound supports more (less) integration, this correlation suggests that subjects traded speed accuracy by controlling the amount of integrated evidence sufficient to make a choice. Using fMRI, these authors showed higher activation in dlPFC under the accuracy condition, and higher activation in the cerebellum under the speed condition. They further considered correlations between activation in each of these regions and that in the striatum (the effective connectivity). Note that the striatum is hypothesized to control response thresholds and thus choice behavior (see below). The effective connectivity between dlPFC and the striatum was higher under the accuracy condition and was positively correlated with the difference (high-low) between the value of the bound parameter under the two conditions. The effective connectivity between the cerebellum and the striatum was higher under the speed condition and was negatively correlated with this difference. Striatal activation did not differ between conditions, consistent with a fixed threshold. Because earlier studies have provided evidence for integrator activity in dlPFC during decisions (Kim and Shadlen, 1999; Heekeren et al., 2004; Philiastides et al., 2011) and for sub-second timing in the cerebellum (see Lewis and Miall, 2003; Ivry and Spencer, 2004), it was hypothesized that persistent changes in connectivity mediate response modes for the purpose of maximizing reward. Thus, the balance between cortico-striatal and cerebellar-striatal processing could control the SAT. This study switched speed and accuracy conditions between blocks, but each block contained very few trials (approximately 10). Subjects therefore adapted quickly to task conditions, suggesting that the underlying mechanism may be capable of switching from trial to trial on cue.

The study by Green et al. (2012) is not the only MRI study to implicate the striatum in the SAT. In the study by Forstmann et al. (2008), the BOLD signal in the pre-supplementary motor area (pre-SMA) and the striatum was stronger in response to a pre-trial cue indicating speed conditions in an RDM task, compared to accuracy or neutral conditions. When individual subject's behavioral data were fit by a bounded accumulator model, the magnitude of adjustments to the bound were positively correlated with the BOLD signal in these areas, i.e., subjects whose behavior was captured by larger adjustments showed greater activation in pre-SMA and striatum. The strength of connectivity between pre-SMA and striatum has also been correlated with individual subjects' adjustments to the bound in an RDM task, i.e., subjects whose behavior was captured by larger adjustments to the bound showed greater connectivity between these areas, as determined by structual MRI (sMRI) (Forstmann et al., 2010).

In the study by Ivanoff et al. (2008), human subjects performed an RDM task with growing motion coherence under speed and accuracy conditions. These authors classified their results according to “baseline trials” and “coherence trials,” where the coherence of moving dots was 0% (over a full trial) and greater than 0% respectively. The underlying premise of this classification is that baseline trials did not provide evidence for integration, but rather, provided only noise; whereas coherence trials provided evidence *and* noise. The BOLD signal in pre-SMA and posterior lateral prefrontal cortex (plPFC) was higher on baseline trials under the speed condition, and was higher on coherence trials under the accuracy condition. Furthermore, the difference in activation under speed and accuracy conditions on baseline trials was equal and opposite to that on coherence trials across subjects, i.e., the speed-minus-accuracy difference on baseline trials equaled the accuracy-minus-speed difference on coherence trials. These data suggest that baseline activity in these cortical regions determines the amount of integrated evidence sufficient to make a choice. In other words, the integrated evidence on coherence trials may account for the difference in activation between speed and accuracy conditions. If so, this equal, opposite difference should be found on a within-subject basis. It was found in pre-SMA, but not in plPFC.

Ivanoff et al. (2008) further showed that on coherence trials, a measure of subjects' decision criteria [the criterion metric of signal detection theory (Macmillan and Creelman, 1991)] was correlated with the BOLD signal in plPFC, but not in pre-SMA. This finding suggests that speed and accuracy conditions modulate the amount of evidence integrated by plPFC. These authors sub-classified their coherence trials according to the level of coherence at the time of subjects' decisions, defining “hits” and “false alarms” as trials on which coherence was positive and 0% at decision time respectively. The BOLD signal in pre-SMA was equal in both classes of trial. Under the assumption that brain regions supporting the integration of evidence should show greater activity on hits than false alarms (because there is evidence to integrate), these data support the hypothesis that evidence is not integrated in pre-SMA. Conversely, activation in plPFC was greater on hits than false alarms, suggesting that plPFC supports integration in the task. Overall, the study by Ivanoff et al. (2008) supports the hypothesis that pre-SMA plays an “adaptive baseline” role in the SAT, determining the amount of evidence integrated in cortical areas such as plPFC. Taken together, the studies by Forstmann et al. (2008), Forstmann et al. (2010), and Ivanoff et al. (2008) suggest that pre-SMA projects non-selectively to the striatum, where this activity is added to selective inputs from cortical integrator populations.

The above studies were extended by van Maanen et al. (2011), who considered the mechanisms by which subjects switch between response modes for speed and accuracy. Under speed conditions, trial-to-trial changes in the BOLD signal in pre-SMA were positively correlated with estimates of the starting point of accumulation in a single-trial version of a bounded accumulator model, in which the bound was fixed. In this case, a higher starting point has the same effect as a lower bound, i.e., faster, less accurate decisions. These data further support the hypothesis that pre-SMA provides a non-selective control signal to the striatum, governing the SAT. On trials that imposed a switch between speed and accuracy conditions (in either direction), a positive correlation was also found between BOLD changes in the anterior cingulate cortex (ACC) and the starting point. Interestingly, only switches from accuracy to speed were correlated with activation of the striatum, suggesting that switching between response modes may be asymmetric, i.e., different mechanisms may mediate switching from a speed mode to an accuracy mode than *vice versa*.

The study by van Maanen et al. (2011) further showed that under accuracy conditions, BOLD changes in ACC were positively correlated with changes in the starting point in their model, but only on trials following an error. These data suggest that ACC may contribute to an emphasis on accuracy, consistent with a neural model of cortico-BG circuitry in which cortical conflict detection excites the subthalamic nucleus (STN) (Frank, 2006; Frank et al., 2007). Note that ACC is believed to play a role in conflict monitoring (Yeung et al., 2004). The model is based on earlier neural models of action selection (Gurney et al., 2001), in which rewards are associated with salient stimuli. In the model by Frank (2006), conflict arises when multiple rewarding (or unrewarding) stimuli occur simultaneously. Cortex detects this “conflict” and projects to STN, which in turn prevents action selection by inhibiting motor circuitry. The model thereby implements dynamic threshold adaptation, increasing the amount of evidence sufficient to make a choice during difficult decisions.

The underlying premises of this “STN hypothesis” (Bogacz et al., 2010b) are further supported by studies of response inhibition in “stop-signal” tasks, in which subjects are cued to withhold planned responses on a proportion of trials (Stop trials). The “direct” and “hyperdirect” pathways have been correlated with Go trials (without the stopping cue) and Stop trials respectively (Aron and Poldrack, 2006), suggesting that activation of the striatum speeds up responding and activation of STN slows it down. These data therefore suggest that speed and accuracy conditions may preferentially activate the direct and hyperdirect pathways respectively (Figure 9A). As described above, speed conditions have been correlated with fronto-striatal circuitry in a number of neuroimaging studies of the SAT (Forstmann et al., 2008; Ivanoff et al., 2008; Forstmann et al., 2010; van Maanen et al., 2011). However, we are unaware of any study to show a positive correlation between STN (activity or connectivity) and accuracy conditions, or a negative correlation between STN and speed conditions. The small size of STN may be a factor in this regard. The present neuroimaging data can therefore be considered to support the notion that accuracy conditions correspond to a “default” mode of decision making, modulated by speed conditions (van Veen et al., 2008; van Maanen et al., 2011). If so, switching between speed and accuracy response modes from trial to trial would only need involve fronto-striatal circuitry, as described above (Forstmann et al., 2008, 2010; Ivanoff et al., 2008). The fMRI data by van Maanen et al. (2011) suggest a more complex state of affairs, but it seems plausible that under this “striatal hypothesis” (Bogacz et al., 2010b), some baseline level of fronto-striatal activation corresponds to a default mode, where speed and accuracy conditions increase and decrease activation respectively.

#### 4.3.3. Adjustments to the connectivity between integrators and thresholding circuitry

The hypothesis that the SAT is supported by adjustments to the connectivity between integrators and thresholding circuitry (Figure 5G) has been implemented in a biophysically-based, coupled-circuit model of eye-movement decisions (Lo and Wang, 2006). In the model, the integration of evidence occurs in cortex and projects directly to the superior colliculus (SC) by excitatory synaptic connectivity, and indirectly via the striatum and substantia nigra pars reticulata (SNr). Note that SC is extensively correlated with eye-movement decisions (e.g., Dorris and Munoz, 1998; Thevarajah et al., 2009). SC is tonically inhibited by SNr, so the latter pathway is disinhibitory. These authors assumed that the pre-saccadic reduction in tonic SNr activity occurs abruptly, rather than smoothly, so SC burst neurons were inactive in the model until SNr was sufficiently inhibited by the striatum. As such, burst neurons detected threshold-crossing by cortical integrator neurons, and consequently, burst firing was much more sensitive to changes in the conductance strength of cortico-striatal synapses than cortico-SC synapses. By tuning the conductance strength of cortico-striatal synapses between blocks of trials, the model traded speed for accuracy. Stronger (weaker) conductance entailed lower (higher) integrator rates under speed (accuracy) conditions, but for a given conductance strength (a given speed/accuracy condition), integrator rates were fixed across task difficulty (Roitman and Shadlen, 2002; Churchland et al., 2008). Note that the model does not appear suited to the trial-to-trial switching of speed and accuracy modes on cue, owing to the timescales of synaptic plasticity.

## 5. Discussion and conclusions

Under the framework of bounded integration, there are three general classes of hypothesis on the neural implementation of the SAT: differential modulation of the encoding of evidence under speed and accuracy conditions (Figure 5A), differential modulation of the integration of encoded evidence (Figures 5B–D), and differential modulation of the amount of integrated evidence sufficient to make a choice (Figures 5E–G). The first category has received the least attention, but the recent study by Heitz and Schall (2012) provides strong evidence for the modulation of sensory encoding (Section 4.1).

Hypotheses on the differential modulation of integration under speed and accuracy conditions can be sub-classified according to the rate (Section 4.2.1) and onset (Section 4.2.2) of integration, and the sensitivity of integrator circuitry to the encoding of evidence (Section 4.2.3). There is considerable evidence for the first of these hypotheses. The rate of rise of putative integrator activity has been shown to increase and decrease under speed and accuracy conditions respectively (Heitz and Schall, 2012). This activity can be explained by attractor models (Figures 3, 7), in which speed (accuracy) conditions increase (decrease) the rate of the evolution of competitive dynamics. At least three neural models have demonstrated that a cognitive signal could control the SAT in this manner by projecting non-selectively to integrator circuitry, either by persistent mnemonic activity (Furman and Wang, 2008; Roxin and Ledberg, 2008) or by climbing activity encoding elapsed time relative to a deadline (Standage et al., 2013).

Hypotheses on the amount of integrated evidence sufficient to make a choice can be sub-classified according to adjustments to non-evidence inputs to integrator circuitry (Section 4.3.1), adjustments of non-integrator inputs to thresholding circuitry (Section 4.3.2) and adjustments to the connectivity from integrator circuitry to thresholding circuitry (Section 4.3.3). According to the first of these hypotheses, if choice behavior requires a fixed level of activity by integrator neurons, then more (less) evidence will be required to reach this fixed level if less (more) common input is provided to all integrators. Attractor models suggest that this mechanism may be impossible to disentangle from the modulation of the rate of integration, since an increase in spatially non-selective excitation decreases their effective time constants, i.e., it increases the rate of integration. Spatially non-selective excitation, however, is not necessarily synonymous with a common input to integrators. The former entails a common input to integrator neurons *and* other neurons in the local circuitry not receiving evidence. The latter does not necessarily include these other neurons. We are unaware of any studies to systematically consider the modulation of recurrent dynamics according to this difference, but the dynamics of attractor networks are known to be influenced by the size of integrator populations relative to the number of neurons in these networks (Albantakis and Deco, 2009).

Our description of the role of BG in the adjustment of non-integrator inputs to thresholding circuitry has not considered bidirectional connectivity between cortex and BG via the thalamus, which complicates the interpretation of information flow during decisions (Figure 9A). The different spatial profiles of cortico-BG-thalamo-cortical loops further complicate things, since information from different cortical areas may be processed discretely within BG and returned to the areas of origin, may be integrated within BG and returned to all regions of origin, or may be partially integrated (see Nambu, 2011). Further to these complications, there are multiple processing pathways though BG. The direct and hyperdirect pathways are described above, but there is also an “indirect” pathway to the output nuclei, via the external segment of the globus pallidus (GPe, Figure 9A). GPe receives inhibitory projections from the striatum and makes inhibitory projections to the output nuclei. The indirect pathway thus “counteracts” the direct pathway, i.e., excitation of the striatum disinhibits motor circuitry along the direct pathway, while effectively inhibiting it via the indirect pathway (dis-disinhibition). Interestingly, STN makes excitatory projections to GPe, so the hyperdirect pathway also has a counteracting pathway, i.e., excitation of STN inhibits motor circuitry, but also disinhibits it via GPe (see Nambu, 2011). Thus, interpreting correlations between SAT behavior and activation of BG input and output nuclei is complicated by the paths this activity may follow, with each path supporting different computations. Extensive discussion of these possibilities is beyond the scope of this review, but assumptions about these and other anatomical factors influence the interpretation of the experimental data presented here.

The possibility of “self-modulation” of decision dynamics (Section 4.3.2) also warrants further comment. The cortico-BG model by Frank (2006) includes a cortical conflict detection area (potentially ACC) that raises the threshold for choice behavior by projecting to STN. Thus, more difficult tasks more strongly activate this area during decisions, raising the threshold. At first glance, this possibility appears to conflict with bounded integrator models in which reward rate is maximized by lowering the bound during decisions (Ditterich, 2006a; Drugowitsch et al., 2012). As noted in Section 4.2.1, lowering the decision criterion reduces time-wasting because it speeds up decisions that are more likely to be wrong, but this approach may not be ideal under stringent accuracy conditions, e.g., when errors are punished by long timeouts. In this case, raising the criterion could be the better strategy. This discrepancy highlights the potential utility of separate mechanisms for speed and accuracy emphasis: it is not immediately clear how a single neural mechanism could implement the within-trial increase in the bound under accuracy conditions and decrease in the bound under speed conditions.

### 5.1. Predictions for future experiments

Different classes of hypothesis on the SAT make different predictions for experimental testing, as do different models within these classes. For instance, the hypothesis that the SAT is controlled by adjustments to non-evidence input to integrator circuitry (Section 4.3.1) makes a different prediction about the rate of integrator activity at the time of commitment to a choice than the hypothesis that the SAT is controlled by adjustments to non-integrator inputs to thresholding circuitry (Section 4.3.2) or adjustments to the connectivity between integrator circuitry and thresholding circuitry. Assuming a fixed current is required for choice selection, adjustments to non-evidence input to integrator circuitry imply the same rate of integrator activity at choice time across task conditions, whereas an increase (decrease) in non-integrator input to thresholding circuitry under speed (accuracy) conditions implies a lower (higher) rate of integrator activity at choice time, as does stronger (weaker) connectivity between these circuits. The only available single-cell data conflict with the latter mechanisms, showing a higher rate of putative integrator activity under speed conditions (Heitz and Schall, 2012). These authors showed that leakage by the circuitry enacting the choice could account for the difference in rate, an explanation that supports the former mechanism.

The conflict between the prediction of lower (higher) integrator rates under speed (accuracy) conditions and electrophysiological data (Heitz and Schall, 2012) raises several points of caution. Firstly, the experimental studies providing evidence for the adjustment of non-integrator inputs to thresholding circuitry employed perceptual tasks in which humans made their choices by manually pressing a button (Forstmann et al., 2008, 2010; Ivanoff et al., 2008; van Veen et al., 2008; Green et al., 2012), whereas the electrophysiological data were recorded during a task in which non-human primates made their choices with an eye-movement. We are comfortable ignoring inter-species differences at this stage of the game, but it is plausible that the pathways from frontal regions to primary motor cortex are qualitatively different in relation to the SAT than those from FEF to eye-movement circuitry (as in Heitz and Schall, 2012). On the other hand, the striatal hypothesis (Section 4.3.2) does not require that non-selective excitation of the striatum be provided by the same cortical area across response modalities. Here, it is worth noting that FEF projects directly to the circuitry mediating eye movements, but also projects to this circuitry along a pathway through the striatum, substantia nigra pars reticulata (SNr) and SC. Because SNr tonically inhibits SC, the latter pathway potentially provides an eye-movement “version” of the striatal hypothesis described above in the context of manual movements. Suffice to say, it would be informative to run the RDM task used by Forstmann et al. (2008) in an eye-movement paradigm.

Different models that account for the SAT by the modulation of the rate of integration (Section 4.2.1) make different predictions about the weighting of evidence during decisions. Stationary attractor models (Furman and Wang, 2008; Roxin and Ledberg, 2008) predict a *primacy effect* (Wong et al., 2007), i.e., earlier evidence is weighted more heavily than later evidence. In effect, attractor dynamics amplify a decision variable, so earlier evidence is subject to amplification for longer. This prediction by stationary attractor models contrasts with that of bounded integrator models dominated by leakage, which show a *recency effect* because earlier evidence is subject to leakage for longer (see e.g., Usher and McClelland, 2001). In time-dependent attractor models (Standage et al., 2011, 2013), if the dynamics are weak at the start of a trial, then a decision variable is dominated by early leakage and late amplification. As such, the evidence will be most heavily weighted somewhere in the middle (see Standage et al., 2011). The respective predictions of these models could be tested by changing the strength of evidence at different times during decision trials. At least one study has conducted such an experiment, using an RDM task in which the coherence of the dots changed during a brief window at different times (Kiani et al., 2008). These authors found a primacy effect, but they used a fixed-duration task with a flat hazard rate, i.e., subjects responded on cue, but it was impossible to determine when the cue would arrive. It would therefore have been impossible to encode elapsed time relative to the cue. Running the same task with a fully predictable duration would be highly informative.

### 5.2. Evidence for limited integration

It is not universally assumed that the neural mechanisms underlying decisions implement the principles of bounded integration as described above (Section 3). In the model by Cisek et al. (2009), momentary evidence is multiplied by elapsed time and a decision is made when the resulting quantity exceeds a decision bound. Because decisions would be susceptible to noise without temporal integration, the authors proposed that noisy evidence is low-passed filtered before being multiplied. A low-pass filter can be thought of as a leaky integrator with a short time constant, so the main difference between this “urgency-gating” model and a leaky integrator with a decreasing bound (see Ditterich, 2006b) is the length of the time constant of integration, i.e., how rapidly the evidence is leaking. Cisek et al. (2009) argued that perceptual decisions in real-world environments are likely to depend on fluctuating evidence, but integrators with long time constants are not well-suited to these conditions. Consistent with these principles, they showed that the urgency-gating model could account for behavioral data from a task with changing evidence, whereas bounded integrator models could not. In effect, the bounded integrators were not leaky enough.

Thura et al. (2012) extended this work by proposing that optimal decisions are supported by the integration of novel information only, where optimality was defined in terms of reward rate. Formally, their model specifies the perfect integration of *differentiated* evidence, where a decision is made when the running total exceeds a decreasing bound. They showed that this procedure is optimal under the assumption of *non*-independence between sequential samples of evidence, which is likely to be the case in most natural conditions, and they proposed that this optimal procedure can be approximated by the multiplication of low-pass filtered evidence by a growing urgency signal. As such, the model is equivalent to their earlier model (Cisek et al., 2009). Their models explain the SAT in tasks with changing evidence because longer (shorter) intervals provide more (less) opportunity to integrate changes in the evidence (novelty). Under the assumption that response-time variability is primarily the result of between-trial variability in attention, arousal and related factors (Carpenter and Williams, 1995), their models further account for behavioral data from traditional tasks with fixed (within-trial) mean evidence, and they account for decision-correlated buildup activity (Section 3.1) under the assumption that this activity mainly reflects the urgency to respond.

In proposing a neural approximation of the urgency-gating model, these authors suggested that the timescale of (leaky) integration is on the order of 100 ms, consistent with evidence that perceptual decisions are based on information from a time window on this order (see Thura et al., 2012), but difficult to reconcile with the SAT on timescales of many hundreds of milliseconds. For example, in the random dot motion task by Palmer et al. (2005), accuracy was lower (higher) and decision times were shorter (longer) under a speed (accuracy) condition, where response times were as long as around 500 ms (2 s). Since the only novel evidence was provided by stimulus onset, the urgency-gating model would appear to predict shorter (longer) decision times under speed (accuracy) conditions, with no change in accuracy, i.e., the integral would have reached its asymptote before 500 ms in either condition, so additional processing time would not improve accuracy. Under the framework of attractor dynamics, however, there is no discrepancy: local-circuit dynamics are subject to modulation, where weak dynamics support a leakage regime and stronger dynamics support a decision regime (Section 3.1.1). As such, modulation of network dynamics by a cognitive signal (Section 4.2.1) can support a range of time constants in the leakage or decision regimes (see Figure 7B). From this viewpoint, cognitive signals projecting to integrator circuitry (or evidence-encoding circuitry) are capable of supporting the effective time constant required by a given context, from around 100 ms (Cisek et al., 2009) to several seconds (Palmer et al., 2005). Under this framework, weak dynamics may be a default mode for decision circuitry under natural conditions (changing evidence), but cognitively demanding tasks may recruit dynamics supporting longer time constants.

The framework of attractor dynamics sheds further light on the possible neural implementation of urgency-gating. A leaky integrator with a short time constant could be implemented by weak local-circuit dynamics, per the first processing stage of Figure 4. In this regard, Thura et al. (2012) noted that the effect of the urgency signal on the decision variables could be additive, not necessarily multiplicative. In the study by Standage et al. (2011), a network model with weak dynamics was subject to gain modulation by a growing urgency signal, i.e., the urgency signal had a multiplicative effect on the decision process. Long time constants were an emergent property of the network, suggesting that a neural implementation of urgency-gating might require additive input. The biophysically-based model by Standage et al. (2013) suggests that this input would need to be spatially selective (targeting each decision variable, but not other local-circuit neurons), since attractor dynamics (with long effective time constants) emerged in their model with a non-selective signal (Section 4.3.1). In principle, the urgency gating model could also be implemented by the projection of the urgency signal to thresholding circuitry, implementing a time-dependent version of the striatal hypothesis (Section 4.3.2) with weak decision dynamics. These and other possibilities require further investigation. Note that there is ample evidence for urgency signals, i.e., climbing activity encoding elapsed time (see Section 4.2.1). The ways in which this activity may modulate decision processing are receiving considerable attention (Ditterich, 2006b; Churchland et al., 2008; Cisek et al., 2009; Hanks et al., 2011; Standage et al., 2011; Drugowitsch et al., 2012; Standage et al., 2013). Changing-evidence tasks represent an important direction in the study of the SAT and decision making more generally.

### 5.3. Distributed integration of evidence and the SAT

The distributed nature of decision processing is an important consideration for all three general classes of hypothesis. For the most part, we have described putative integrator activity one cortical area at a time [e.g., dlPFC (Kim and Shadlen, 1999), LIP (Roitman and Shadlen, 2002; Thomas and Pare, 2007) and FEF (Ding and Gold, 2012; Heitz and Schall, 2012)], highlighting the rate of this activity at choice time in a given electrophysiological experiment. It is likely that different decision-correlated cortical areas encode different dimensions of a given task. Changes to the profile of activity in these areas may therefore differ with task conditions. For example, a higher (lower) rate of FEF movement neurons under speed (accuracy) conditions (Heitz and Schall, 2012) may be accompanied by a lower (higher) rate of activity in dlPFC and/or LIP. All three areas project (at least indirectly) to the circuitry driving eye-movements. The distributed nature of decision processing is well-appreciated by researchers in decision neuroscience, but it is often implicit in electrophysiological studies (and studies based on electrophysiological data) that the relevant decision variable is the one being recorded. There are good reasons for choice thresholds to be fixed (see Marshall et al., 2012), but a fixed choice threshold need not imply a fixed rate of decision-selective activity in each of the brain regions projecting to the relevant motor circuitry. Rather, the aggregate input to the motor circuitry may be fixed, with varying contributions from upstream areas in different conditions.

To further complicate matters, decision-correlated brain areas are often bidirectionally coupled (e.g., FEF, LIP, and SC), so these areas presumably modulate each other during decisions. It is therefore plausible that in a given area, spike rates may indeed be fixed at the time of commitment to a choice, but that peak rates reflect the post-decision dynamics of choice behavior (see Simen, 2012). In light of these considerations, there is a need for electrophysiological recordings from multiple decision-correlated areas under speed and accuracy conditions, e.g., dlPFC, LIP, and/or FEF during eye-movement tasks. The different ways in which decision variables in these areas are modulated by speed and accuracy conditions will not only be informative about the contributions of these areas to the SAT, but also about the roles they play in decision making more generally.

Similarly, the decision dynamics described above (Section 3.1.1) are based on single-circuit models, i.e., local-circuit integration of inputs from upstream, evidence-encoding neurons. We are unaware of any neural modeling studies to systematically consider the dynamics of bidirectionally-coupled decision circuits. It is clear that single-circuit attractor models cannot provide a full account of decision making. For example, these models necessarily produce longer error trials than correct trials (see Wong and Wang, 2006; Standage et al., 2011), but correct trials are longer under some task paradigms (see Ratcliff and Smith, 2004).

### 5.4. A unifying perspective

We have described the above hypotheses one at a time, largely in isolation from one another, but as indicated in Section 4, these hypotheses should not be considered mutually exclusive. The electrophysiological data by Heitz and Schall (2012) are revealing in this regard, providing evidence for the modulation of sensory encoding, the rate of integration and the strength of non-evidence inputs to integrator circuitry. These data are consistent with the hypothesis that a cognitive signal projects non-selectively to sensory-encoding populations *and* integrator populations, controlling the SAT by gain modulation. Such a signal could be implemented by dlPFC (van Veen et al., 2008; Wenzlaff et al., 2011). It is possible that such a signal also projects to thresholding circuitry. Unlike non-selective input to integrator circuitry, which controls integration times in attractor models (Furman and Wang, 2008; Roxin and Ledberg, 2008; Standage et al., 2013), non-selective input to thresholding circuitry may have a negligible effect on local-circuit dynamics, given the already-strong dynamics hypothesized to support the implementation of thresholds (Simen, 2012). Such a cognitive signal could also project to pre-motor regions [e.g., pre-SMA (Forstmann et al., 2008, 2010; Ivanoff et al., 2008)], raising their baseline rates, in turn lowering motor thresholds for choice behavior. This description of the SAT assumes that the cognitive signal is present in neutral conditions, where its rate increases and decreases under speed and accuracy conditions respectively. This hypothesis unifies much of the data presented above and is depicted in Figure 9B.

Despite the long history of behavioral data describing the SAT, these are early days in its mechanistic study (Bogacz et al., 2010b). Recent electrophysiological (Heitz and Schall, 2012), neuroimaging (Forstmann et al., 2008, 2010; Ivanoff et al., 2008; van Veen et al., 2008; Wenzlaff et al., 2011; Green et al., 2012; Ho et al., 2012) and biophysically-based modeling (Lo and Wang, 2006; Furman and Wang, 2008; Standage et al., 2013) studies are exemplary of the promising methods being brought to bear on this fundamental cognitive phenomenon.

### Conflict of interest statement

The authors declare that the research was conducted in the absence of any commercial or financial relationships that could be construed as a potential conflict of interest.
